# Development of a green metallochromic indicator for selective and visual detection of copper(II) ions

**DOI:** 10.1038/s41598-023-39556-x

**Published:** 2023-08-02

**Authors:** Mehran Minabi-Nezhad, Farid Moeinpour, Fatemeh S. Mohseni-Shahri

**Affiliations:** Department of Chemistry, Bandar Abbas Branch, Islamic Azad University, Bandar Abbas, 7915893144 Iran

**Keywords:** Analytical chemistry, Environmental chemistry, Organic chemistry

## Abstract

Heavy metal ions, i.e., copper(II) (Cu(II)), are harmful to the environment and our health. The current research established an eco-friendly and efficient metal-sensitive indicator, which can identify Cu(II) ions in both liquid and solid forms, by utilizing anthocyanin extract obtained from jambolao fruit (*Syzgium cumini*) that is incorporated within bacterial cellulose nanofibers (BCNF).The CIE Lab color parameters demonstrated that Cu(II) binding causes a sensible change in color. It was observed that the visible color altered with an increase in the Cu(II) concentration. The bacterial cellulose nanofibers that were altered with anthocyanin were analyzed using ATR-FTIR and FESEM. The sensor's selectivity was tested by using a range of metal ions such as lead (Pb^2+^), cobalt (Co^2+^), cadmium (Cd^2+^), nickel (Ni^2+^), aluminium (Al^3+^), barium (Ba^2+^), manganese (Mn^2+^), zinc (Zn^2+^), mercury (Hg^2+^) and sodium (Na^+^). The findings demonstrated that the suggested sensor showed excellent selectivity toward Cu(II) ion. Cu(II) can be accurately identified using the sensing technique, with detection limits ranging from 10–400 ppm and 50–500 ppm for liquid and solid samples, respectively, and through observation with naked eye. The fabricated green metallochromic sensor is promising to be a simple, cheap, mobile and easily operable for the real-time and on-site detection of Cu(II) ion.

## Introduction

Copper(II) ions are important minerals that make up a balanced food regimen and are found in dietary supplements and can lead to serious illnesses such as dyslexia and hypoglycemia^1^. Numerous industries release Cu(II) ions in their production processes. It can enter the food chain and collect withinside the human body^2–4^. Copper ions are common contaminants produced by corrosion of domestic piping systems and erosion of natural sediments, and can cause gastrointestinal disturbances, as well as liver and kidney damage. Therefore, they are one of the important heavy metal ions^5^. The development of highly sensitive and selective chemical sensors for the detection of Cu(II) ions is urgently needed due to its potential adverse effects on environment and the health of the people. The examination of heavy metal ions can be executed using different methods, including atomic absorption spectrometry and coupled plasma mass spectrometry^6^, anodic stripping voltammetry^7,8^, inductively coupled plasma mass spectrometry^9^, microprobes^10^ and X-ray fluorescence spectrometry^11^. While the methods discussed here have proven to be successful, their usage is restricted due to their advanced technological requirements, time-consuming nature, destructive sampling techniques, and expensive preparation procedures. Amid the various identification methods accessible today, there has been significant focus on colorimetric sensors owing to their simplicity and aptitude for on-site observation. It was discovered that various detectors, predominantly color-based, incorporated TMB-Ag(I) arrangements, two- and three-dimensional ordered super microporous monoliths^12^, silver nanoparticles^13^, gold nanomaterials, and so on^14^. Despite their effectiveness, chemicals are harmful, hence it is crucial to explore environmentally safe sensor substances, such as natural pigments.

Anthocyanins are water-soluble pigments present in plants, fruits, and vegetables that impart a reddish, bluish, or purplish hue to them^15^. They are also part of a group of chemicals known as flavonoids, which possess antioxidant characteristics and have garnered substantial attention in the domain of food technology due to the advantageous effects they offer^16^. Under acidic conditions, anthocyanin has a tendency to appear red, while under neutral conditions it exhibits a blue or purple hue, and under alkaline conditions it displays green or yellow tones. Naturally present food items such as red cabbage, radish, black carrots, grapes, eggplant, and other colorful foods contain anthocyanins, but the tropical fruit jambolao (*Syzygium cumini*) has a variety of colors. Jambolao extract's primary ingredient, anthocyanin, has strong antioxidant properties. The small, ovoid jambolao fruit, which belongs to the *Myrtaceae* family and is indigenous to India, ripens to a purple red to black color due to the high anthocyanin content^17,18^.

**Figure 1:**
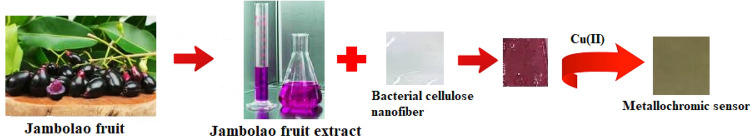
Diagrammatic representation of the BCNF-ANT sensor fabricated for the identification of Cu(II).

The most prevalent biopolymer found in nature is cellulose^19^. Based on the structure, techniques and roles of cellulose, three primary classifications can be identified as: cellulose nanofibers (CNFs), cellulose nanocrystals (CNC), and bacterial cellulose nanofibers (BCNF). Nanofibers made of cellulose from bacteria that are not harmful, *Acetobacter xylinum*, have emerged as a highly promising biomaterial. These nanofibers have demonstrated immense potential in the advancement of paper sensors. A bacterial nanocellulose sheet has been chosen as a proficient base for the creation of a novel range of paper-oriented sensors owing to its exceptional chemical and mechanical features, advantageous biological traits (compatibility with living organisms, biodegradability), permeable structure, see-through nature, and ability to be printed on^20^. A range of optical nanoparticles, either luminescent or colorimetric, can be incorporated into the BCNF nano-network scaffold, which is three-dimensional and porous^21^. BCNF, an economical and eco-friendly material, can serve as a base when combined with reactive substances and is extensively utilized in fields related to energy storage and sensing ^22^. The primary benefits of BCNF as a base material include its ability to be renewed, its biodegradability, its biocompatibility, as well as its possession of flexible shapes, strong mechanical properties, a large specific surface area, and ultrafine fibers^23,24^. Furthermore, the permeable lattice framework is advantageous for the permeation and amalgamation of dynamic components on BCNF to produce purposeful detecting substances, expanding the utilization of BCNF, and presenting a fresh perspective for creating adaptable sensors. One aspect of BCNF's superior mechanical performance is attributed to the well-organized arrangement of crystal domains that result from the plentiful presence of hydrogen bonding^25^. Conversely, robust intramolecular and intermolecular hydrogen bonds create a tightly-knit network structure for BCNF, rendering it resistant to dissolution^26^. Consequently, the progress in utilizing BC-oriented practical sensing substances is restricted, and it is imperative to have viable production methods to devise and enhance BCNF-derived sensing substances. The growth of sensors based on cellulose is gaining extensive and growing interest, and cellulose has been employed to fabricate physical, biological, and chemical sensors separately or in conjunction with other substances^27^. Using the interplay of anthocyanin phenolic pigment and Cu(II) in a liquid state, along with the 3D nano-network scaffold of the BCNF sheet, this research has designed an uncomplicated and biocompatible detector that alters color upon exposure to Cu(II) ions. The platform has been elucidated with respect to both its selectivity and detection limit. The detection of Cu(II) ions can be accomplished with high efficiency, rapidity, simplicity and cost-effectiveness. The schematic representation of colorimetric sensing Cu(II) using anthocyanin bacterial cellulose nanofiber (BCNF-ANT) depicted in Fig. 1.

## Experimental

### Materials

The investigation was conducted utilizing damp BCNF sheets obtained from Nano Novin Polymer Co. situated in Sari, Iran. From the local market, the fruits of jambolao were purchased (Bandar Abbas, Iran). The fruits were cleaned by submerging them in a sodium hypochlorite solution (100 g/mL) for 10 min. They were then washed in distilled water and allowed to air dry on perforated trays for two hours at 30 °C. Using stainless steel knives, the pulp skin-the edible portion of jambolao fruits-was separated from the seeds, frozen at -18 °C, and then shielded from light. Metallic salts such as lead nitrate (Pb(NO_3_)_2_), cobalt nitrate (Co(NO_3_)_2_), nickel sulfate (NiSO_4_), aluminum chloride (AlCl_3_), barium chloride (BaCl_2_), manganese sulfate (MnSO_4_), cadmium chloride (CdCl_2_), copper nitrate trihydrate (Cu(NO_3_)_2_.3H_2_O), zinc chloride (ZnCl_2_), mercury chloride (HgCl_2_), and sodium nitrate (NaNO_3_) were provided by Merck. Adequate quantities of these salts were dissolved in deionized water to create stock solutions. Initially, the copper(II) nitrate stock solution was produced at a concentration of 1000 parts per million (ppm), after which gradual dilutions were made (10–1000 ppm). Solutions of acetate buffer were made in the lab by mixing sodium acetate and glacial acetic acid. The other substances (of analytical grade) were supplied by Sigma-Aldrich.

### Extraction of anthocyanin

According to the process outlined by Brito et al., anthocyanin used in the creation of the films was extracted from jambolao fruits^18^. Jambolao fruits (5 g) were added to HCl 1.5 M/ ethanol 95% (15:85, v/v) solvent mixture, left to stand for 14 h at 5 °C without being exposed to light. After soaking, the mixture was vacuum-filtered, and the leftover material was then washed with the same solvent in order to extract the most anthocyanins possible, up to a volume of 100 mL. To prepare for analysis, the anthocyanin extracts were put into amber flasks and kept at 5 °C.

### Instruments

The UV–visible spectrophotometer (SPEKOL 1500) was used to measure the absorbance within the range of 300 to 700 nm. Thermo Nicolet 370 (Thermo Fisher, the USA) was employed to record the ATR-FTIR spectra. The spectral range registered was between 4000 and 400 cm^−1^, with an interval of 2 cm^−1^. The SEM images were obtained using FESEM (Field Emission Scanning Electron Microscopy) (TESCAN, Czech Republic).

### Characterization of anthocyanin

A Shinoda test was performed to determine the presence of flavonoids. The test involved adding the anthocyanin extract to a methanolic extract (2 to 3 mL) and observing if it produced a red color, using a piece of magnesium ribbon and HCl (1 mL)^28^.

### Provision of BCNF-ANT (anthocyanin cellulose nanofiber)

For a duration of 1.5 h at ambient temperature with gentle agitation (50 revolutions per minute), a total of 5 BCNF sheets (measuring 20 × 2.5 × 0.3 cm^3^) were submerged in a solution containing 100 mL of anthocyanin. In order to eliminate the un-captured anthocyanins from the BCNF-ANT sheets subsequent to their separation from the solution, multiple rinses were conducted with deionized water for the purpose of eliminating the anthocyanins from the sheets. Clasp clips were employed to press the BCNF-ANTs between two glass sheets coated with regular filter paper (Whatman). Following a duration of two hours at a temperature of 100°C, the glass slides were extracted from the oven. To implement the BCNF-ANTs, the dehydrated BCNF-ANTs were conserved in a desiccated brown receptacle.

### Analysis of the Cu(II) ions by colorimetry

The assessment of Cu(II) analyte through colorimetry and visual recognition was performed by utilizing anthocyanin extracted solution and produced BCNF-ANT film at room temperature. The test involved the addition of Cu(II) ions at particular concentrations ranging from 10 to 1000 ppm. pH was adjusted at ~ 7.0. Each of the copper solutions with varying concentrations received an addition of 50 μL of anthocyanin. Additionally, aqueous solutions with diverse metal salts such as Pb(NO_3_)_2_, Co(NO_3_)_2_, CdCl_2_, NiSO_4_, AlCl_3_, BaCl_2_, Cu(NO_3_)_2_.3H_2_O, MnSO_4_, ZnCl_2_, HgCl_2_, and NaNO_3_ were prepared and evaluated at a concentration of 400 ppm. Various coloration measurements were recorded after submerging the BCNF-ANT sensing membrane in every aqueous solution. After the time response 5 min, the absorbance intensities of the samples was measured at 516 nm by UV–vis spectrophotometer. As for the film, a piece of the film (BCNF-ANT) was immersed in the aqueous solutions containing different metal salts for 5 min and then dried. The hue of the gathered specimen was evaluated through visual observation and UV–visible spectroscopy at a wavelength of 516 nm.

### Coloration measurements

The CIE Lab established parameters were used to monitor the colorimetric changes using a freeware version of ImageJ® (http://imagej.nih.gov/ij). The calculation of ΔE values was done as follows^29^:

1 $$\Delta E=\sqrt{{({L}^{*}-{L}_{0}^{*})}^{2}+{({a}^{*}-{a}_{0}^{*})}^{2}+{({b}^{*}-{b}_{0}^{*})}^{2}}$$

where, *L*_0_^*^, a_0_^*^ and *b*_0_^*^ were the numeral values of lightness (white to black), color ranges from red to green, and color ranges from yellow to blue, respectively gathered at 0 min, and L^*^, a^*^ and b^*^ were the values at the time for sampling.

## Results and discussion

### Characterization of anthocyanin and BCNF‑ANT films

Shinoda's assay was utilized to ascertain the existence of flavonoids in the commodity, which resulted in the development of a crimson hue within a short span of time.

### ATR-FTIR spectroscopic investigation

**Figure 2:**
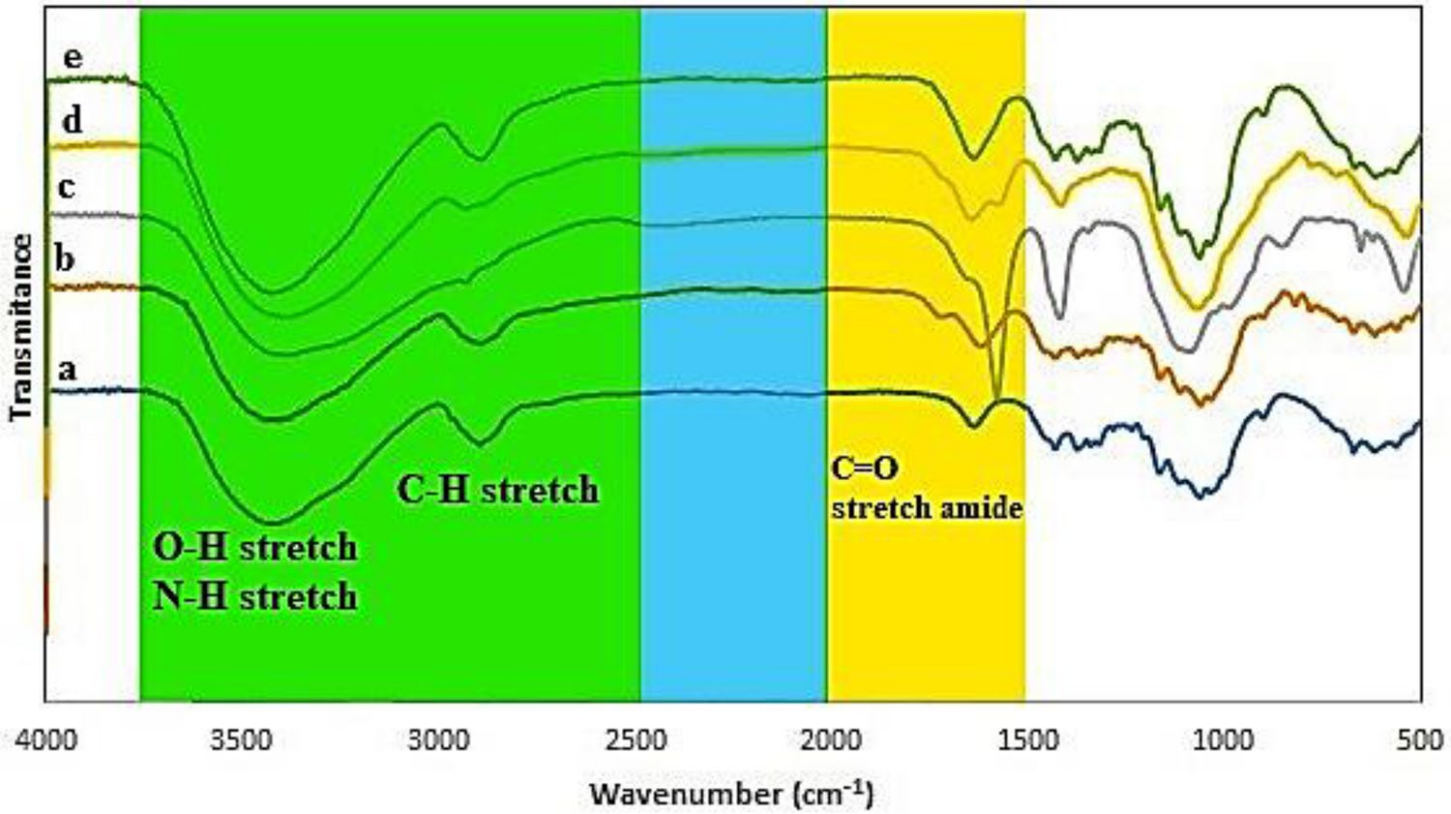
ATR**-**FTIR spectrum of (**a**) BCNF, (**b**) BCNF-ANT**,** (**c**) BCNF-ANT-Cu(II), (**d**) diluted ANT and (**e**) diluted ANT- Cu(II). White, yellow, blue and green areas refer to the fingerprint region, double bonds, triple bonds and single bond stretch respectively.

Through the utilization of ATR-FTIR spectroscopy, the chemical composition of the biopolymer/polymer materials was determined, alongside the existence or non-existence of distinct functional units. Essentially, there are two main forms of functional units: hydroxyl and amine. The vibrations (and spectral location) of these functional units can be modified by interactions with other substances^30^. The spectra of BCNF, BCNF-ANT, BCNF-ANT-Cu (following the adsorption of Cu(II) ion), ANT diluted and diluted ANT-Cu(II) (a to e) are presented in Fig. 2 using ATR-FTIR.

The property bands of BCNF are as follows: the amide-A group's hydrogen coupling causes the O–H and N–H stretching at 3341 cm^−1^^31^, the C-H stretching vibration at 2894 cm^−1^, and the C–C stretching vibration, skeletal vibrations, and ring vibrations at 1426, 1315, 1158, 1104, and 1032 cm^−1^, respectively.

Anthocyanins were detected in BCNF films through the identification of peaks related to the stretching of aromatic rings (1600–1584;1500–1400 cm^−1^). The amide-I band detected in the samples at 1630 cm^−1^ can be accounted for by the stretching of C=O in BCNF carboxyl groups. The observed alterations in the peaks at ~ 3500–3200 cm^−1^ and ~ 1650–1500 cm^−1^ indicate that hydrogen bonds were formed between the O–H groups of anthocyanin and the O–H and N–H groups of BCNF^32^. The Cu(II) and BCNF-ANT interaction caused a shift in the C=O peak at 1600 cm^−1^, affirming that the binding of Cu(II) ions with anthocyanin involves C=O groups^33^.

The diluted ANT spectrum (Fig. 2d) exhibits a prominent absorption band at 1650 cm^−1^, which corresponds to the stretching vibration of the C=C aromatic ring. Upon comparing the spectra of diluted ANT-Cu(II) and diluted ANT, it is evident that the absorption band at 1650 cm^−1^ is more pronounced in the former (Fig. 2e). In general, upon addition of Cu(II) ions to the mixture, the strength of the stated band reduced.

### Morphological characteristics of BCNF‑ANT films

**Figure 3:**
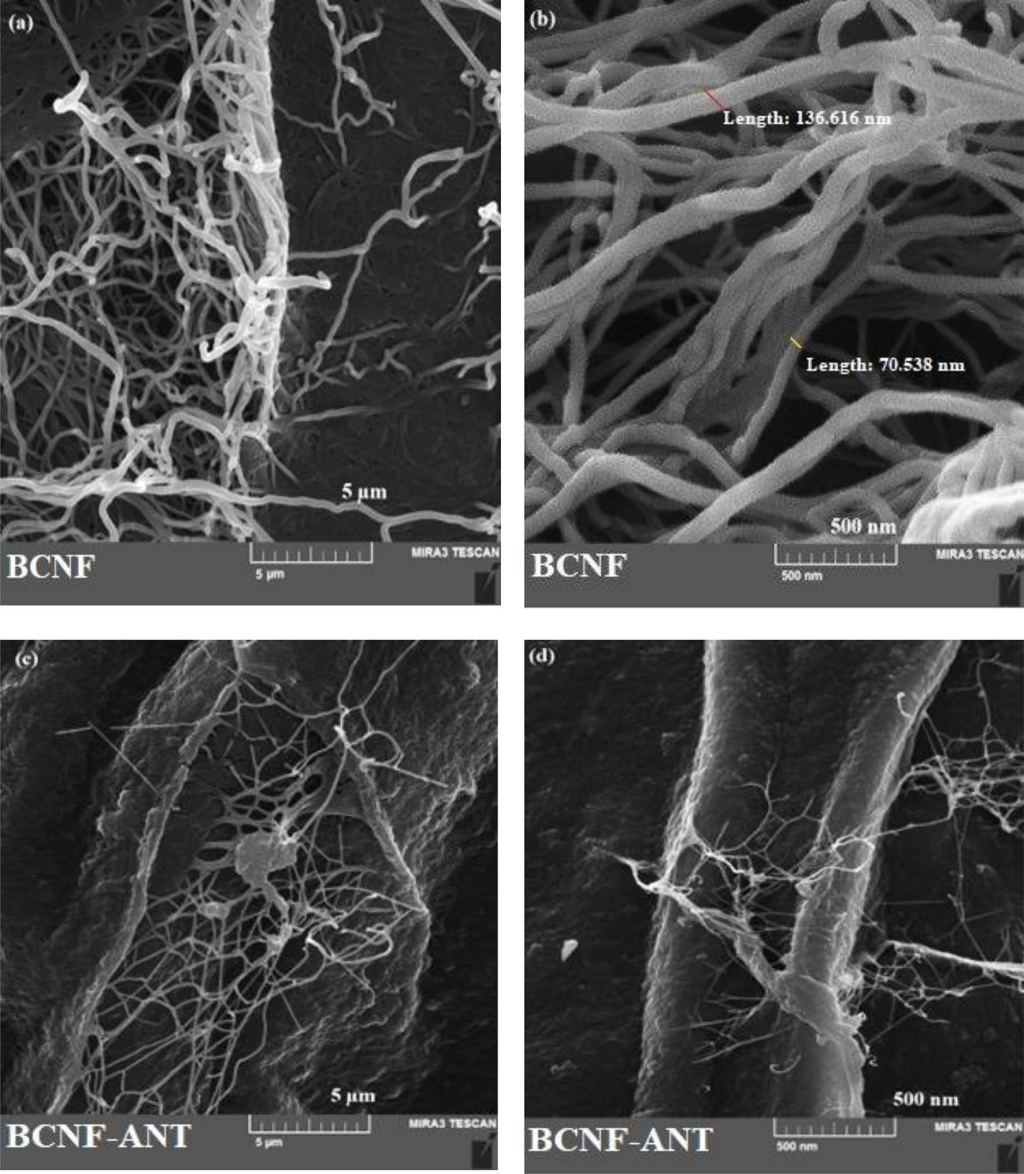
FESEM pictures of (**a**, **b**) BCNF and (**c**, **d**) BCNF-ANT with a 5 µm and 500 nm scale bar.

The SEM images were used to study the entrapment of anthocyanin extract in the BCNF film. Figure 3 illustrates SEM images of BCNF and BCNF-ANT as per the given guidelines. The comparison between the two SEM micrographs (Fig. 3a and c) with a scale bar of 5 µm revealed that anthocyanin was effectively incorporated into the clear nano-network of BCNF.

As indicated by a 500 nm scale bar, both surfaces exhibit a miniaturized nanofibrous morphology of BCNF.

### Colorimetric performance of anthocyanin solution and BCNF‑ANT film

**Figure 4:**
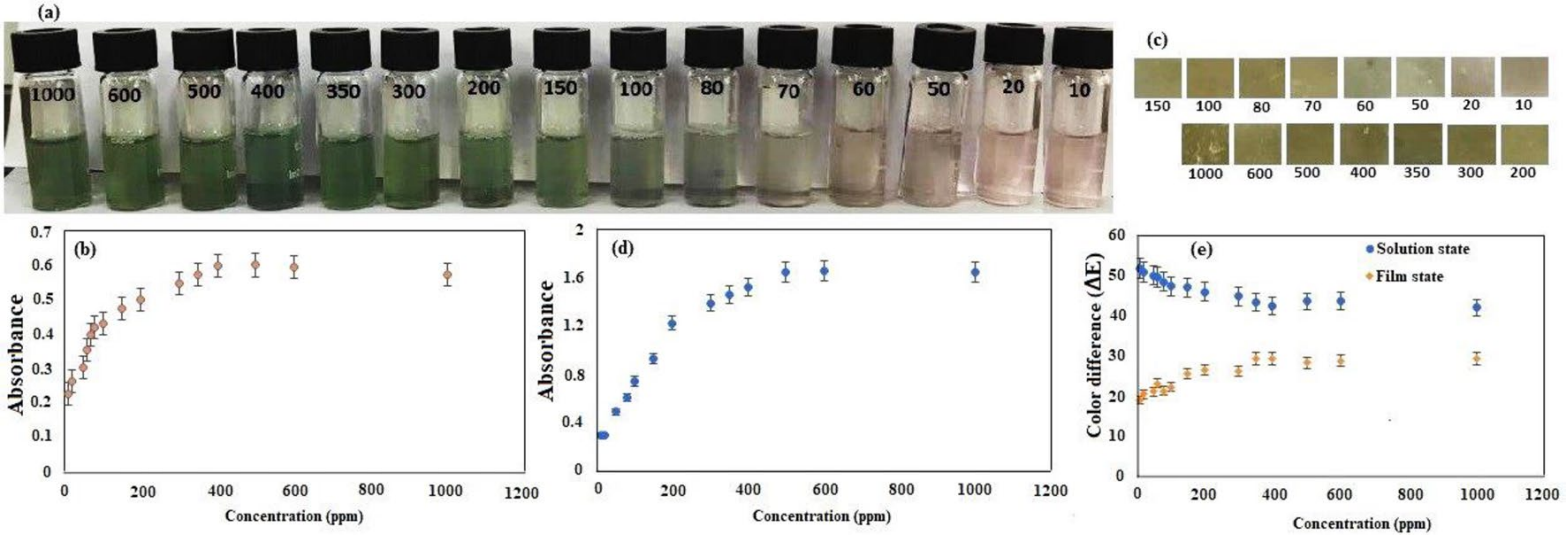
Color variation of (**a**) anthocyanin solution and (**c**) BCNF-ANT sensor in different concentration (10–1000 ppm) of Cu(II), changes in absorbance intensity of (**b**) anthocyanin solution and (**d**) BCNF-ANT sensor at 516 nm upon sensing the increased concentration (10–1000 ppm) of Cu(II) in aqueous medium, (**e**) total color difference (ΔE) of anthocyanin solution and BCNF-ANT sensor in different concentration (10–1000 ppm) of Cu(II).

As depicted in Fig. 4a, in a solution state, the visible color alterations range from light pink to deep green as the concentration of Cu(II) ions rises from 10 to 1000 ppm. The UV–vis spectrum of the produced BCNF‑ANT membrane displayed a distinctive peak at λ = 516 nm, linked to the existence of ANT, which verified the successful development of the BCNF‑ANT membrane (Fig. S1).

Proof suggests that the creation of coordination bonds among phenolic anthocyanins and Cu(II) results in the development of a range of green-colored complexes, contingent on the concentration of copper. The comprehensive amount of Cu(II) in aqueous form displayed a correlation outline (Fig. 4b).

At a wavelength of 516 nm, the absorbance intensities displayed a correlation pattern that was directly proportional to the rise in Cu(II) levels ranging from 10 to 1000 ppm. Specifically, the absorbance intensities rose in tandem with the increase in Cu(II) concentration. When the aqueous concentration of Cu(II) was decreased to less than 10 ppm, there were no alterations in the absorption strength of the solution at 516 nm. Consequently, a detection limit of under 10 parts per million (ppm) was observed. The rise in Cu(II) concentration led to the identification of noticeable colorimetric alterations. The results suggested that augmenting the Cu(II) level to 400 ppm did not result in a surge in the intensity of the absorption peak at 516 nm. Therefore, to determine the detection limit, the copper concentration was raised until it attained 400 ppm.

As indicated by Fig. 4c, the BCNF-ANT film can be distinguished by its observable hue when subjected to Cu(II) levels that vary from 10 to 1000 ppm. The intensities of absorption at 516 nm are depicted in Fig. 4d. It exhibited a minimal detectable level of 50 ppm and a maximum level of 500 ppm. It is noteworthy that the analysis of the image was carried out in conformity with Eq. 1 and the values of ΔE indicated in Fig. 4e. In general, the colorimetric characteristics of the indicator were evaluated using ΔE and any alterations in color were noticeable to the naked eye if ΔE exceeded 5^34^. The findings suggest that the indicator exhibited ΔE values greater than 15 when the concentrations varied from 10 to 1000 ppm.

### BCNF-ANT film and anthocyanin solution's selectivity for Cu(II) ions

**Figure 5:**
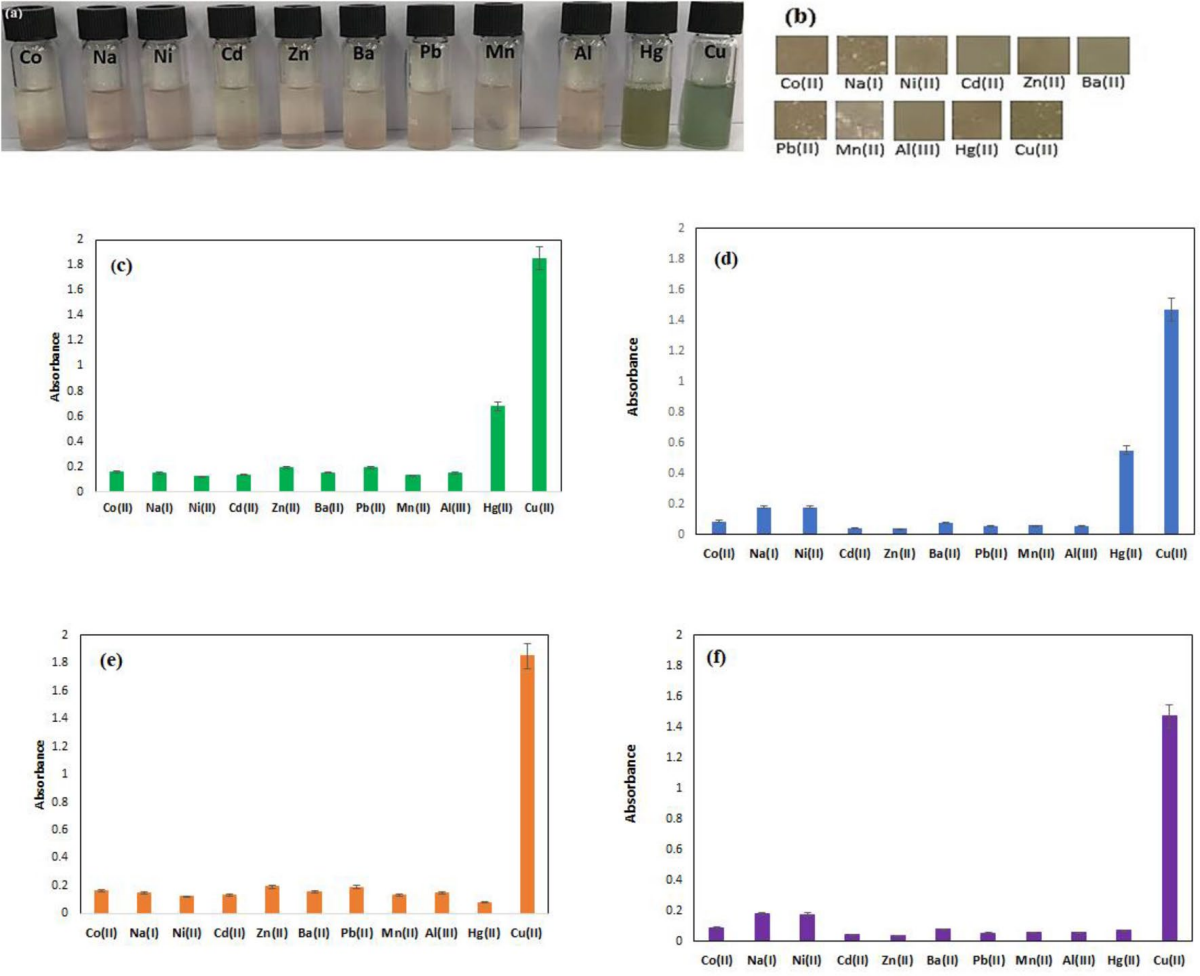
Selectivity evaluation of the (**a**) anthocyanin solution and (**b**) BCNF-ANT sensor (400 ppm), comparative insight on the intensities of the absorption maxima for (**c**) anthocyanin solution, (**d**) BCNF-ANT sensor, (**e**) anthocyanin solution with SCN^-^ and (**f**) BCNF-ANT sensor with SCN^-^ at 516 nm for different metal cations (400 ppm).

The specificity of the anthocyanin solution and BCNF-ANT film for Cu(II) ions was examined in the presence of various chosen metal ions, including Pb^2+^, Co^2+^, Zn^2+^, Ni^2+^, Al^3+^, Ba^2+^, Cd^2+^, Hg^2+^, Mn^2+^, and Na^+^. According to the absorption readings at 516 nm, it was evident that the anthocyanin solution exhibited a significant preference for Cu(II) ions (Fig. 5a). A relative perception regarding the absorption intensities of anthocyanin solution at 516 nm for the designated metal ions (at constant concentration; 400 ppm) is illustrated in Fig. 5c.

Moreover, the selectivity of BCNF-ANT sensor was examined in the presence of metal ions. Each ion was used at a concentration of 400 ppm and the BCNF-ANT sheets were incubated for 5 min. As depicted in Fig. 5b and d, a noticeable disparity in color was observed between the selected metal ions and Cu(II). The alteration in color of BCNF-ANT occurred due to the binding of anthocyanin with Cu(II).

Only minor variations in the CIE Lab colorimetric parameters (L^*^, a^*^, and b^*^) were found by examining the BCNF-ANT sensor in the presence of metal ions (Table 1). Hg(II), like Cu(II), however, displayed a small amount of sensitivity to the BCNF-ANT sensor. The color strength of the BCNF-ANT sensor in solution and film state was improved in the presence of Cu(II) ions. In addition, the a^*^ value varied from positive to negative, indicating a change in hue from red to green, while the positive b^*^ value increased, indicating a change in hue from a more bluish hue to a more yellowish one^35,36^. Nevertheless, metallic cations (Cu(II) and Hg(II)) are frequently present together in diverse ecological and physiological specimens. Consequently, masking agents were employed to attain the selective concurrent identification of every metallic element while in the presence of another metallic element. SCN- functions as a masking agent for Hg(II) ions, as it exhibits a higher stability constant with Hg(II) ions and displays limited coordination ability with Cu(II) ions^37,38^. As illustrated in Fig. 5e and f, ANT and BCNF-ANT effectively formed a complex with Cu(II) ion, even in the existence of Hg(II) ion, with the utilization of SCN^−^ as a masking agent.

**Table 1:** Color coordinates for BCNF-ANT sensor upon the detection of some selected metal ions at a constant concentration (400 ppm).

Metal ions	Solution state			Film state		
	L*	a*	b*	L*	a*	b*
Co(II)	56.615 ± 1.3	2.02 ± 0.04	10.927 ± 0.47	51.616 ± 1.34	2.143 ± 0.04	15.453 ± 0.15
Na(I)	53.956 ± 0.95	4.545 ± 0.13	9.886 ± 0.57	56.764 ± 1.12	2.060 ± 0.14	16.294 ± 0.27
Ni(II)	53.484 ± 0.87	3.702 ± 0.11	8.805 ± 0.44	54.459 ± 0.98	1.928 ± 0.23	16.651 ± 0.61
Cd(II)	55.132 ± 1.32	1.638 ± 0.04	10.979 ± 0.75	59.489 ± 0.78	1.404 ± 0.08	16.561 ± 0.73
Zn(II)	58.209 ± 0.76	2.892 ± 0.02	10.352 ± 0.82	54.417 ± 1.02	0.938 ± 0.02	17.770 ± 0.38
Ba(II)	55.701 ± 0.46	2.854 ± 0.11	11.199 ± 0.51	51.043 ± 0.87	0.669 ± 0.01	17.648 ± 0.48
Pb(II)	55.801 ± 0.88	2.415 ± 0.21	11.013 ± 0.89	52.846 ± 0.47	− 0.133 ± 0.03	17.575 ± 0.17
Mn(II)	55.174 ± 0.79	1.793 ± 0.06	9.207 ± 0.22	55.089 ± 0.57	− 0.252 ± 0.05	17.416 ± 0.24
Al(III)	53.663 ± 0.36	3.210 ± 0.05	11.045 ± 0.48	54.042 ± 1.21	− 0.417 ± 0.04	17.761 ± 0.31
Hg(II)	37.796 ± 0.27	− 5.049 ± 0.14	17.692 ± 0.61	49.963 ± 1.34	− 1.108 ± 0.07	18.987 ± 0.67
Cu(II)	42.096 ± 0.41	− 8.811 ± 0.23	12.081 ± 0.51	49.466 ± 0.83	− 1.647 ± 0.06	21.261 ± 0.21

### Sensor sensitivity to pH

**Figure 6:**
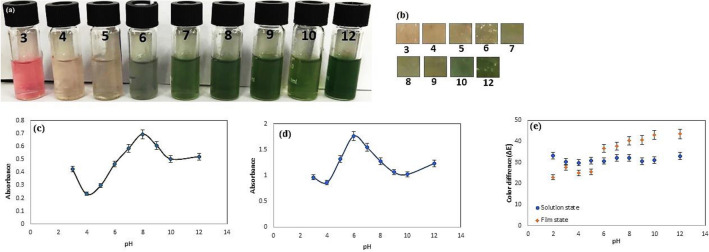
Color variations of (**a**) anthocyanin solution and (**b**) BCNF-ANT sensor (Cu(II) 400 ppm) at different pHs, changes in absorbance intensity of (**c**) anthocyanin solution and (**d**) BCNF-ANT sensor at 516 nm upon sensing Cu(II) (400 ppm) in aqueous media at various pHs, (**e**) the ΔE values in various pHs.

Changes in pH affect anthocyanins. So, 400 ppm Cu(II) was prepared as a stock aqueous solution. Buffer solutions containing acetate were added to increase the pH value from 3 to 12. The color changes of anthocyanin solution and BCNF-ANT sensor (containing Cu(II) 400 ppm) at different pHs are shown in Fig. 6a and b, respectively. UV-Vis absorbances were recorded for the solutions prepared at different pH levels. Fig. 6c, d shows the graph of the UV-Vis absorbance intensity versus the pH value in solution and film state respectively.

The UV–vis absorbance intensity increases as the pH increases from 4 to 8 in the solution state. It then decreases until pH 10, which suggests a high range of potential binding between Cu(II) ions and anthocyanin active sites. The reason for this can be credited to the numerous -OH groups present on the molecular structure of anthocyanin, which can function as sites of coordination. Any variations in the intensity of UV–vis absorbance become negligible when the pH exceeds 10.

In film state, when the pH rises from 4 to 6, the intensity of UV–vis absorbance increases, but subsequently declines until it reaches pH 10. The maximum absorption intensity was at pH 8 and 6 in solution and film, respectively. Hence, fabricated sensor can be utilized as a selective sensor for Cu(II) ions in a wide range of pH 4.0–10.0.

At a pH of 6, the anthocyanin carried a negative charge while the copper existed predominantly as CuOH^+^ and Cu(OH)_2_^39^. Simultaneously, the biopolymers (BCNF) underwent deprotonation^40,41^, resulting in the electrostatic affinity between the surface of the film and the copper components, thereby aiding the creation of the ANT-Cu complex^42–44^. Similar results with other metals have been obtained by other researchers^45,46^.

The total color change of the sensor was computed by color parameters of the sensor at pH 3–12. As seen in Fig. 6e, ΔE values in all pHs were greater than 5, which illustrated a good color variation of the indicator at acidic to basic conditions.

### Real sample measurement

**Figure 7:**
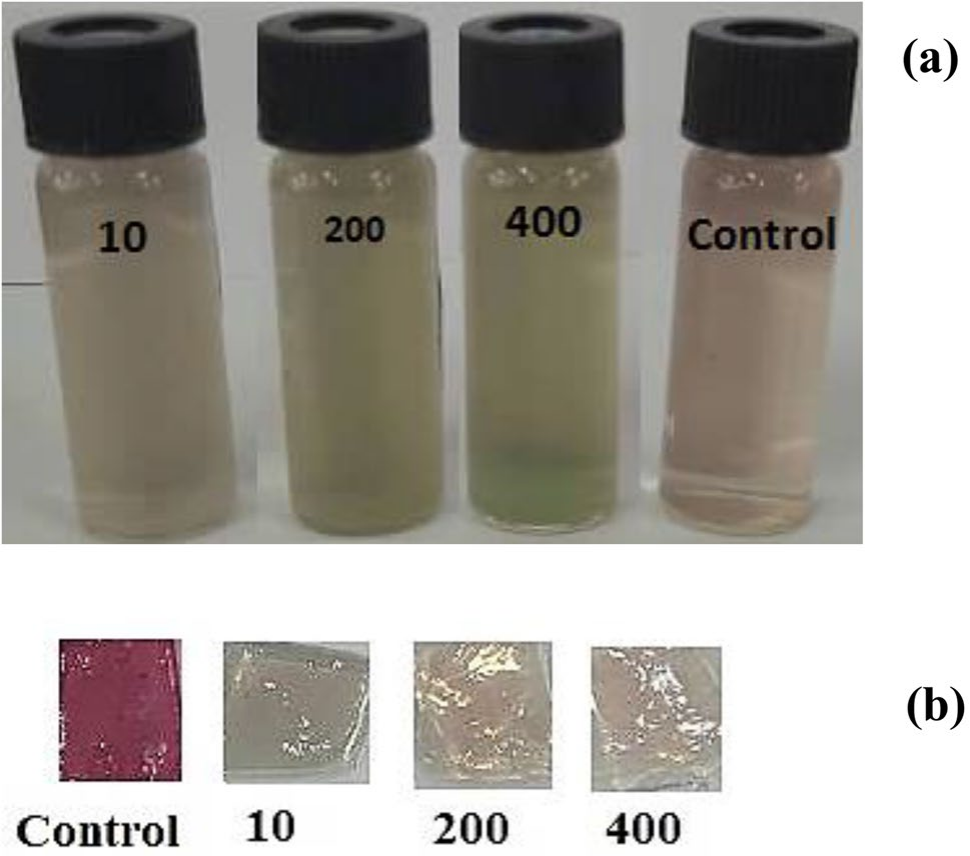
The pictures of the real samples (tap water) spiked by the various concentrations of Cu(II) ion (10, 200 and 400 ppm), (**a**) anthocyanin solution and (**b**) BCNF-ANT sensor.

The surveillance of copper (II) ions in fresh water is also important, as water offers an alternative route for copper (II) to penetrate the living organism systems. To identify the presence of copper ions in an actual sample, varied concentrations of Cu(II) solutions were introduced to the tap water sample. This was done to attain a final concentration of 10, 200, and 400 ppm of Cu(II) in the sample. Figure 7 displays the related images. The calculated ΔE values for the water sample obtained from the tap are exhibited in Table 2. All the ΔE values were greater than 5, suggesting that the alterations in color could be easily discerned by the unaided eye when compared to the original color of both the solution and the film. The results suggested that the fabricated sensor could effectively be utilized in the analysis of actual samples.

**Table 2:** The ΔE values for the real sample analysis.

Concentration of Cu(II) (ppm)	10	200	400
ΔE value (solution state)	6.122 ± 0.45	9.066 ± 0.37	8.938 ± 0.93
ΔE value (film state)	32.937 ± 0.38	33.256 ± 0.62	34.646 ± 0.58

Recent studies have demonstrated that colorimetric sensing systems are effective, user-friendly, and cost-efficient detection tools for metallic ions. The anthocyanin extract may serve as a sensor for Cu(II) ions, exhibiting exceptional specificity, responsiveness, and eco-friendliness. This research revealed that jambolao fruit anthocyanins can serve as a portable solid-state and solution-state sensor for identifying Cu(II) ions. The detection limit in solution state ranges from 10 to 400 ppm, while in film state, it ranges from 50 to 500 ppm.

Ethylenediaminetetraacetic acid (EDTA) was additionally employed for the extraction of Cu(II) ions from the utilized BCNF-ANT film to assess the sensor's recyclability. Nonetheless, as a result of the nanofiber arrangement of bacterial cellulose, it was unfeasible to entirely eliminate the assimilated Cu(II) particles within a rational duration.

Table 3 juxtaposes the analytical proficiency of our sensor with that of other sensors utilizing synthetic chemicals as molecular probes. Evidently, our sensor possessed a lesser sensitivity compared to the sensors utilizing synthetic molecular probes. However, this type of sensor is highly effective for detecting Cu(II) in water in situ, without the use of organic solvents, making it a simple and environmentally safe option for self-monitoring water. Unlike the sensors mentioned previously, the colorimetric sensor produced by this research does not necessitate any electronic components, skilled individuals, or sophisticated equipment for its usage.

**Table 3:** Comparison of previously reported Cu(II) detection sensors with the BCNF-ANT.

Sensor	Methos of detection	LOD (ppm)	Reference
Calix[4]arene based	Colorimetric	0.0001	^47^
Polymer inclusion membrane	Colorimetric	0.06	^48^
HMPAMB*	Colorimetric	0.00034	^49^
Metal–organic frameworks (UiO-66)	Colorimetric	0.00049	^50^
BCNF‑ANT	Colorimetric	50	This study

*HMPAMB = 2,2′ -((1E,1′ E)-((6-hydroxy-2-mercaptopyrimidine-4,5-diyl) bis(azaneylylideneazaneylylidene))bis(methaneylylidene)) bis(4-bromophenol).

## Conclusion

We created an uncomplicated, speedy, inexpensive, particular, responsive, and mobile color-changing sensor that detects Cu(II) ions both in liquid and solid states using metallochromic technology. The identification method mainly relied on anthocyanin obtained from jambolao fruit as a spectroscopic chromatic element immobilized on a bacterial cellulose nanofibrous (BCNF) host in solid state. The sensing technique can accurately identify Cu(II) with detection limits ranging from 10–400 ppm and 50–500 ppm in solution and solid phases, correspondingly. We developed a sensor that can detect Cu(II) ions in aqueous matrices in the pH range from 4.0 to 10.0, both in solution and film state. The sensor is capable of producing a visual color change that depends on the Cu(II) concentration.

The sensor we developed is highly selective in the presence of different metal ions, including Pb^2+^, Co^2+^, Zn^2+^, Ni^2+^, Al^3+^, Ba^2+^, Cd^2+^, Hg^2+^, Mn^2+^ and Na^+^. The real tap water sample was effectively treated using a solution of anthocyanin and a BCNF-ANT sheet. Our colorimetric sensor does not require electronic components, trained personnel, or sophisticated equipment. It can be used on-site to monitor Cu(II) contamination in food matrices.

## Supplementary Information

Supplementary Information.

## Data Availability

All data produced or examined throughout this research are incorporated in this published work.
