# Supplementary material for: Development of a green metallochromic indicator for selective and visual detection of copper(II) ions
Source: Sci Rep. 2023 Aug 2;13:12501. doi: 10.1038/s41598-023-39556-x (PMC10397238; doi:10.1038/s41598-023-39556-x)
Supplement: Supplementary file 1 — Supplementary Information. [file 41598_2023_39556_MOESM1_ESM.doc]

**Electronic Supplementary Information (ESI) for**

**Development of A Green Metallochromic Indicator for Selective and Visual Detection of Copper(II) Ions**

Mehran Minabi-Nezhad^a^, Farid Moeinpour^a*^, Fatemeh S. Mohseni-Shahri^a^

^a^ Department of Chemistry, Bandar Abbas Branch, Islamic Azad University, Bandar Abbas 7915893144, Iran

^*^Corresponding author.

Farid Moeinpour, Department of Chemistry, Bandar Abbas Branch, Islamic Azad University, Bandar Abbas 7915893144, Iran. Email: [fmoeinpour@iauba.ac.ir](mailto:fmoeinpour@iauba.ac.ir)


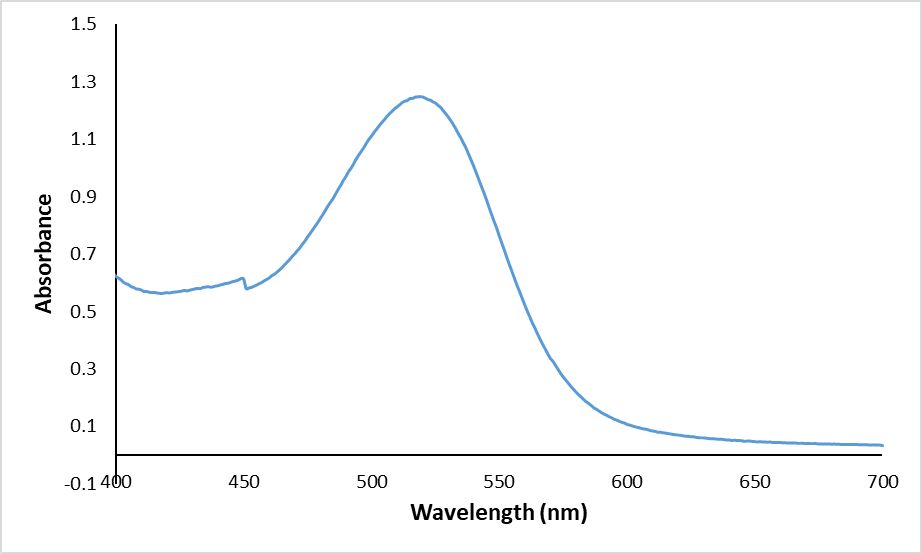


**Fig. S1.** UV-vis spectrum of the fabricated BCNF‑ANT.
